# Supplementary material for: The mitotic spindle is chiral due to torques within microtubule bundles
Source: Nat Commun. 2018 Sep 3;9:3571. doi: 10.1038/s41467-018-06005-7 (PMC6120957; doi:10.1038/s41467-018-06005-7)
Supplement: Supplementary file 1 — Supplementary Information [file 41467_2018_6005_MOESM1_ESM.pdf]

Supplementary Information

The mitotic spindle is chiral due to torques within microtubule bundles

Novak et al.

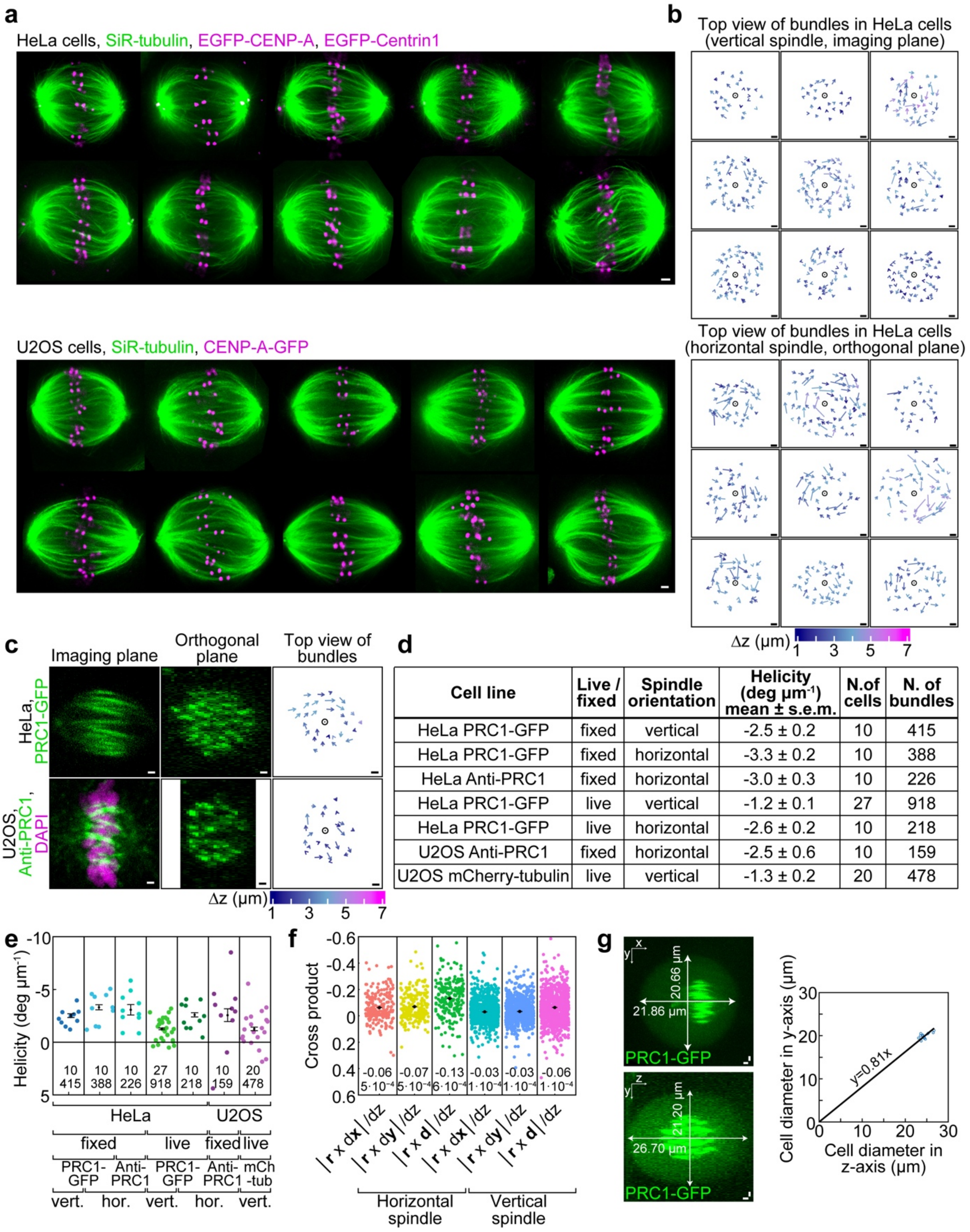

Cell line	Live / fixed	Spindle orientation	Helicity (deg μm-1) mean ± s.e.m.	N.of cells	N. of bundles
HeLa PRC1-GFP	fixed	vertical	-2.5 ± 0.2	10	415
HeLa PRC1-GFP	fixed	horizontal	-3.3 ± 0.2	10	388
HeLa Anti-PRC1	fixed	horizontal	-3.0 ± 0.3	10	226
HeLa PRC1-GFP	live	vertical	-1.2 ± 0.1	27	918
HeLa PRC1-GFP	live	horizontal	-2.6 ± 0.2	10	218
U2OS Anti-PRC1	fixed	horizontal	-2.5 ± 0.6	10	159
U2OS mCherry-tubulin	live	vertical	-1.3 ± 0.2	20	478

Supplementary Figure 1. Extended characterization of spindle chirality

a, STED images of metaphase spindles in live HeLa cells expressing EGFP-CENP-A and EGFP-centrin1 (magenta) (top), and live U2OS cells expressing CENP-A-GFP (magenta) (bottom). Microtubules are labeled with SiR-tubulin (green). **b**, Arrows connecting starting and ending points of bundles from fixed HeLa cells expressing PRC1-GFP and mRFP-CENP-B,

traced upwards, from vertical (top) and horizontal (bottom) spindles. Circle denotes spindle major axis. Z-distance between the first and last tracked point of a bundle is color coded, see color bar. **c**, Top, imaging plane of a horizontal spindle in a live HeLa cell expressing PRC1-GFP, with DNA labelled by SiR-DNA (only PRC1-GFP is shown) (left); orthogonal plane of the same spindle (middle); arrows connecting starting and ending points of PRC1-GFP bundles traced upwards (right). Longer arrows roughly correspond to larger twist around the spindle axis (circle), colors show z-distance between starting and ending points, see color bar. Bottom, horizontal spindle in an unlabeled U2OS cell immunostained for PRC1, with DNA stained by DAPI, legend as in the top row. Left, maximum intensity projections of 5 central planes; middle, single planes. **d**, Summary of spindle helicity values for conditions and cell lines as indicated. Cell lines used: HeLa cells expressing PRC1-GFP, unlabeled HeLa cells immunostained for PRC1, unlabeled U2OS cells immunostained for PRC1, U2OS cells expressing CENP-A-GFP, mCherry-α-tubulin and photoactivatable (PA)-GFP-tubulin. **e**, Spindle helicity averaged over cells for different conditions and cell lines as in **d**. Each dot represents a weighted mean over all bundles in a cell. Weight was determined by the number of points tracked in a bundle. The numbers in each column represent the number of cells (top) and bundles (bottom), error bars, s.e. with respect to the weighted mean. **f**, Cross product between the normalized position vector and the components of the total bundle displacement vector, as well as with the displacement vector itself. Bundles from all untreated live HeLa cells expressing PRC1-GFP are shown. r, normalized position vector; dx, displacement in x direction; dy, displacement in y direction; d, total displacement. Displacement in x-direction (dx) and y-direction (dy) are similar, suggesting there was no anisotropy in image transformation. Numbers in each group denote the mean (top) and s.e.m. (bottom). **g**, xy projection of a live HeLa cell expressing PRC1-GFP, with horizontally oriented spindle (top); the same cell in yz projection (bottom); linear regression showing the relationship between the cell diameter in y and z axes (right). Ratio of y and z diameter was 0.814±0.008 (mean±s.e.m., n=13). This ratio was different from 1 due to refractive index mismatch, and was used as a correction factor for z-coordinates (see Methods). Ratio of x and y diameter, 0.99±0.01, was not different from 1. All scale bars, 1 μm.

a

Cell type	Treatment	Helicity (deg µm^{-1}) mean ± s.e.m.	Number of cells	Number of bundles	p-values
HeLa horizontal	None	-2.6 ± 0.2	10	218	7·10^{-11}
HeLa horizontal	STLC (5-25 min)	-0.9 ± 0.2	16	317	
HeLa vertical	None (0 min)	-1.2 ± 0.1	27	918	2·10^{-9}; 2·10^{-6}
HeLa vertical	STLC (5 min)	-0.2 ± 0.2	12	395	
HeLa vertical	STLC (10 min)	-0.2 ± 0.2	8	203	
HeLa vertical	None (0 min)	-1.2 ± 0.1	27	918	0.042; 0.33
HeLa vertical	DMSO (5 min)	-0.9 ± 0.2	10	337	
HeLa vertical	DMSO (10 min)	-1.0 ± 0.2	7	228	
U2OS vertical	None	-1.3 ± 0.2	20	478	3·10^{-6}
U2OS vertical	STLC (10 min)	0.01 ± 0.2	8	141	
HeLa horizontal	None	-2.6 ± 0.2	10	218	0.41
HeLa horizontal	Lat A (1-2 hours)	-2.4 ± 0.2	10	240	

b

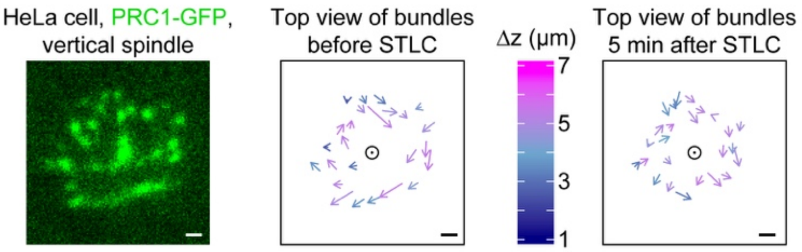

c

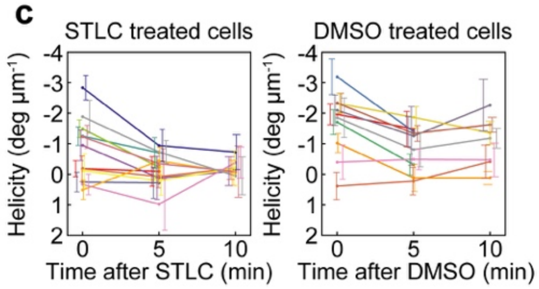

d

Treatment	Spindle dimensions (µm), mean ± s.e.m.	Number of cells	p-values
STLC	Length = 11.5 ± 0.2	16	0.79
None	Length = 11.6 ± 0.4	10	
STLC	Width = 9.4 ± 0.2	16	0.34
None	Width = 9.1 ± 0.2	10	

Supplementary Figure 2. Extended analysis of spindles after treatments with STLC and latrunculin A

a, Summary of spindle helicity values from live cells treated with STLC, Latrunculin A or DMSO in different conditions as indicated. **b**, Equatorial plane of a vertical spindle in a live untreated HeLa cell expressing PRC1-GFP (left); arrows connecting starting and ending points of PRC1-GFP bundles traced upwards, from the same cell, before (middle) and 5 minutes after STLC treatment (right). Circle denotes spindle axis. Z-distance between the first and last traced point is color coded, see color bar. **c**, Helicity of vertical spindles from individual live HeLa cells expressing PRC1-GFP measured before and at 5 and 10 minutes after treatment with either STLC (left) or DMSO (right). Values from each cell are represented by different color and connected with lines. **d**, Spindle length and width from horizontal live HeLa cells expressing PRC1-GFP treated with STLC or untreated, as indicated. Error bars, s.e.m. p-values, unpaired t-test. Scale bars, 1 µm.

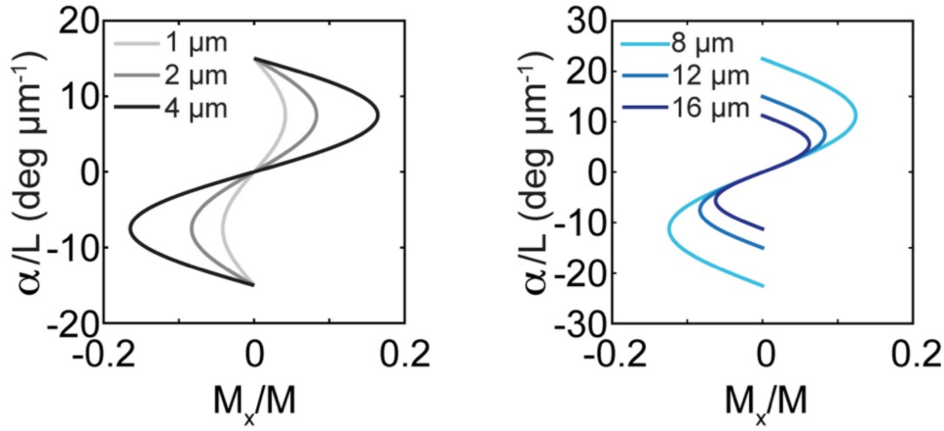

Supplementary Figure 3. Additional results from the model
Twist of a microtubule bundle, α, divided by spindle length, L, as a function of the twisting moment, $M_x = M_{ix}$, normalized to the bending moment, $M = \sqrt{M_{iy}^2 + M_{iz}^2}$ (the same curve for $i = 1{,}2$). Results are shown for three sizes of the spindle pole, $2d = 1, 2$ and 4 μm (left), and three lengths of the mitotic spindle, $L = 8, 12$ and 16 μm (right). The other parameters are $L =$ 12 μm (left) and $d = 1$ μm (right), whereas $\kappa = 900$ pN μm^2.
