# Supplementary material for: The mitotic spindle is chiral due to torques within microtubule bundles
Source: Nat Commun. 2018 Sep 3;9:3571. doi: 10.1038/s41467-018-06005-7 (PMC6120957; doi:10.1038/s41467-018-06005-7)
Supplement: Supplementary file 3 — Description of Additional Supplementary Files [file 41467_2018_6005_MOESM3_ESM.docx]

**Description of Additional Supplementary Files**

**File Name: Supplementary Movie 1**

**Description:**

PRC1-labeled microtubule bundles rotate around the spindle axis

Scheme of imaging a vertically oriented spindle (left), gray frame denotes imaging plane. Images of a vertical spindle from a fixed HeLa cell expressing PRC1-GFP and mRFP-CENP-B (right, only PRC1-GFP is shown). Distance between z-planes is 500 nm; scale bar, 1μm.

**File Name: Supplementary Movie 2**

**Description:**

Tracking of microtubule bundles in vertically oriented spindle

Examples of tracking of two PRC1-GFP bundles in the same spindle from Supplementary Movie 1. Tracking points are denoted by numerated empty circles. First and last point in the first tracked bundle (points 1 and 15) denote spindle poles. Planes containing tracking points and text were duplicated twice and four times, respectively, to improve readability. Distance between z-planes is 500 nm; scale bar, 1μm.

**File Name: Supplementary Movie 3**

**Description:**

Three-dimensional reconstruction of the tracked bundles

Dots connected by lines represent traced bundles from Supplementary Movie 1. Colors represent different bundles. Coordinate system is represented as a cuboidal box. Black line represents spindle major axis.

**File Name: Supplementary Movie 4**

**Description:**

Horizontal spindle from a fixed HeLa cell expressing PRC1-GFP and mRFP-CENP-B

Scheme of a horizontal spindle with the imaging plane (top left) and the orthogonal plane (bottom left). z-stack of a horizontal spindle from a fixed HeLa cell expressing PRC1-GFP and mRFP-CENP-B, only PRC1-GFP is shown for clarity (top right). Reconstructed images of the same spindle after transformation (rotation) into vertical orientation (bottom right). Distance between imaged z-planes is 500 nm; scale bars, 1μm.

**File Name: Supplementary Movie 5**

**Description:**

Horizontal spindle from an unlabeled HeLa cell immunostained for PRC1

Z-stack of a horizontal spindle from a fixed unlabeled HeLa cell immunostained for PRC1 (left). Reconstructed images of the same spindle after transformation (rotation) into vertical orientation (right). Chromosomes were labeled with DAPI to identify metaphase (not shown). Distance between z-planes is 500 nm; scale bar, 1μm.

**File Name: Supplementary Movie 6**

**Description:**

Horizontal spindles from a live HeLa cell expressing PRC1-GFP, untreated and treated with STLC

Z-stack of an untreated horizontal spindle from a live HeLa cell expressing PRC1-GFP (top left). Reconstructed images of the same spindle after transformation (rotation) into vertical orientation (top right). z-stack of an STLC-treated horizontal spindle from a live HeLa cell expressing PRC1-GFP (bottom left). Reconstructed images of the same STLC-treated spindle after transformation (rotation) into vertical orientation (bottom right). Chromosomes were labeled with SiR-DNA to identify metaphase (not shown). Distance between z-planes is 500 nm; scale bars, 1μm.

**File Name: Supplementary Movie 7**

**Description:**

Tracking of microtubule bundles in a vertically oriented spindle of a live U2OS cell expressing mCherry-α-tubulin and CENP-A-GFP

Examples of tracking of two microtubule bundles in a spindle from a live U2OS cell expressing mCherry-α-tubulin (green) and CENP-A-GFP (magenta). Tracking points are denoted by numerated empty circles. First and last point in the first tracked bundle (points 1 and 12) denote spindle poles. Planes containing tracking points and text were duplicated twice and four times, respectively, to improve readability. Distance between z-planes is 500 nm; scale bar, 1 μm.

**File Name: Supplementary Movie 8**

**Description:**

Horizontal spindle from an unlabeled U2OS cell immunostained for PRC1

Z-stack of a horizontal spindle from a fixed unlabeled U2OS cell immunostained for PRC1 (left). Reconstructed images of the same spindle after transformation (rotation) into vertical orientation (right). Chromosomes were labeled with DAPI to identify metaphase (not shown). Distance between z-planes is 500 nm; scale bar, 1μm.
